# COVID-19-associated Guillain-Barré syndrome in the early pandemic experience in Lombardia (Italy)

**DOI:** 10.1007/s10072-022-06429-6

**Published:** 2022-10-26

**Authors:** Filippo Martinelli-Boneschi, Antonio Colombo, Nereo Bresolin, Maria Sessa, Pietro Bassi, Giampiero Grampa, Eugenio Magni, Maurizio Versino, Carlo Ferrarese, Davide Zarcone, Alberto Albanese, Giuseppe Micieli, Carla Zanferrari, Antonio Cagnana, Claudio Ferrante, Angelo Zilioli, Davide Locatelli, Maria Vittoria Calloni, Maria Luisa Delodovici, Mattia Pozzato, Valerio Patisso, Francesco Bortolan, Camillo Foresti, Barbara Frigeni, Stefania Canella, Rubjona Xhani, Massimo Crabbio, Alessandro Clemenzi, Marco Mauri, Simone Beretta, Isidoro La Spina, Simona Bernasconi, Tiziana De Santis, Anna Cavallini, Michela Ranieri, Elisabetta D’Adda, Maria Elisa Fruguglietti, Lorenzo Peverelli, Edoardo Agosti, Olivia Leoni, Andrea Rigamonti, Andrea Salmaggi

**Affiliations:** 1 Neurology Unit, IRCCS Fondazione Ca’ Granda Ospedale Maggiore Policlinico, Via Francesco Sforza 35, 20122 Milan, Italy; 2 Department of Health Sciences, University of Milan, Via Antonio di Rudinì 8, 20142 Milan, Italy; 3 Membro Direttivo Nazionale SNO, Polo Neurologico Brianteo, Seregno, MB Italy; 4 U.O. Neurologia Ospedale Giovanni XXIII, Bergamo, Italy; 5 U.O. Neurologia, Ospedale San Giuseppe, Milan, Italy; 6 U.O. Neurologia, Ospedale Sant’Anna, Como, Italy; 7 U.O. NeurologiaPoliambulanza, Brescia, Italy; 8 Università Dell’ InsubriaU.O. Neurologia Ospedale Di Varese, Varese, Italy; 9 Università Degli Studi Milano Bicocca, U.O. Neurologia, Ospedale San Gerardo, Monza, Italy; 10 U.O. Neurologia, Ospedale Sant’Antonio Abate, Gallarate, VA Italy; 11 U.O. Neurologia, IRCCS Istituto Clinico Humanitas, Milan, Italy; 12 U.O. Neurologia, Fondazione Mondino, Pavia, Italy; 13 U.O. Neurologia, Ospedale Vizzolo Predabissi, Vizzolo Predabissi, MI Italy; 14 U.O. Neurologia, Ospedale Maggiore, Crema, CR Italy; 15 U.O. Ospedale Policlinico Ponte San Pietro, Ponte San Pietro, BG Italy; 16 U.O. NeurologiaOspedale Maggiore, Lodi, Italy; 17 Università Insubria, U.O. NeurochirurgiaOspedale Di Varese, Varese, Italy; 18 Membro Direttivo Nazionale SNO, Rome, Italy; 19 Membro Direttivo Regionale Lombardo SNO, Milan, Italy; 20 U.O. Osservatorio Epidemiologico Regionale, Struttura Epidemiologia E Valutazione Delle Performance, Milan, Regione Lombardia Italy; 21 U.O. Neurologia, Ospedale Manzoni, Lecco, Italy; 22 Coordinatore SNO Lombardia, U.O. Neurologia, Ospedale Manzoni, Lecco, Italy

**Keywords:** Guillain-Barré syndrome, COVID-19, *SARS-CoV-2* infection, Coronavirus infections

## Abstract

**Objective:**

To estimate the incidence and describe clinical characteristics and outcome of GBS in COVID-19 patients (COVID19-GBS) in one of the most hit regions during the first pandemic wave, Lombardia.

**Methods:**

Adult patients admitted to 20 Neurological Units between 1/3–30/4/2020 with COVID19-GBS were included as part of a multi-center study organized by the Italian society of Hospital Neuroscience (SNO).

**Results:**

Thirty-eight COVID19-GBS patients had a mean age of 60.7 years and male frequency of 86.8%. CSF albuminocytological dissociation was detected in 71.4%, and PCR for *SARS-CoV-2* was negative in 19 tested patients. Based on neurophysiology, 81.8% of patients had a diagnosis of AIDP, 12.1% of AMSAN, and 6.1% of AMAN. The course was favorable in 76.3% of patients, stable in 10.5%, while 13.2% worsened, of which 3 died. The estimated occurrence rate in Lombardia ranges from 0.5 to 0.05 GBS cases per 1000 COVID-19 infections depending on whether you consider positive cases or estimated seropositive cases. When we compared GBS cases with the pre-pandemic period, we found a reduction of cases from 165 to 135 cases in the 2-month study period in Lombardia.

**Conclusions:**

We detected an increased incidence of GBS in COVID-19 patients which can reflect a higher risk of GBS in COVID-19 patients and a reduction of GBS events during the pandemic period possibly due to a lower spread of more common respiratory infectious diseases determined by an increased use of preventive measures.

## Introduction

Coronavirus disease 2019 (COVID-19), caused by severe acute respiratory syndrome coronavirus 2 (*SARS-CoV-2*), has rapidly evolved into a worldwide pandemic. Based on data from the World Health Organization (WHO) (https://covid19.who.int/), as of January 22, 2022, around 340.6 million cases have been diagnosed and 5.5 million died globally. In Lombardia, a region in the Northern part of Italy of 10 million inhabitants, 19.1% of the population has been infected (compared to 16.1% in Italy), with a lethality of 1.9% which is higher than the Italian (1.5%) and global one (1.6%) (https://lab24.ilsole24ore.com/coronavirus/#). We recall that in the first pandemic wave in 2020, as shown by several documents, 8.362/13.710 (61%) deaths in Italy due to COVID-19 infection concerned Lombardy [1].

COVID-19 predominantly affects the respiratory tract and lung parenchyma, but there is evidence of neurological involvement by COVID-19. A study from China reported that 36.4% of patients had neurological symptoms, including headache, dizziness, impaired smell and taste, encephalopathy, acute cerebrovascular disease, skeletal muscle injury, as well as impairment of peripheral nervous system (PNS) [2], and another study from France reported that 84% of patients with severe COVID-19 presented with neurological sequelae [3].

Moreover, several studies reported an increased incidence of Guillain–Barre’ syndrome (GBS) after various epidemics around the world like the Zika epidemic in French Polynesia [4], and also after *SARS-CoV* and Middle East respiratory syndrome virus (MERS) infections [5].

More recently, numerous case report/series and cross-sectional studies have described cases of GBS linked to *SARS-CoV-2* infection, which suggests a possible association between GBS and COVID-19. The first reported case of GBS was diagnosed in late January 2020 in an otherwise asymptomatic COVID-19 patient who developed COVID-19 symptoms at day 8 of GBS [6]. The first series of 5 COVID-19 patients who later developed GBS was reported in April 2020 by an Italian Group [7]. A systematic review has conducted an individual participant data (IPD) meta-analysis and performed meta-regression after assessing the quality of the included articles including 61 cases extracted from 45 articles from 16 different countries [8]. Another complete review of cases with GBS and COVID-19 was performed, including 73 patients from 52 publications [9].

In the present study, we estimate the incidence and describe the clinical characteristics and outcome of GBS in COVID-19 patients (COVID-GBS) in the first pandemic period (March 1 to April 30, 2020) in one of the most affected regions by COVID-19 of the world, Lombardia, as part of a cross-sectional study which included 20 Neurology Units of the region. As a second aim of the study, we compared the incidence of GBS during the pandemic period in Lombardia in the same 2-month period between March and April in pre-pandemic period (2019) and pandemic period (2020 and 2021) to evaluate a fluctuation of GBS cases before and during pandemic.

## Methods

A multi-center, cross-sectional study on neurological complications in patients with COVID-19 was organized by the Italian society of Hospital Neuroscience (SNO) including 20 Neurology Units in Lombardia which was the most affected Italian region during the first wave of COVID-19 pandemic, and it is nowadays in the fourth wave. The general aim of the cross-sectional study was to assess clinical features, treatment, and outcomes of several neurological complications involving the central nervous system (CNS) (ischemic and hemorrhagic acute cerebrovascular disease, encephalitis, encephalopathy, cerebral venous sinus thrombosis) and the peripheral nervous system (PNS), including GBS. All cases underwent a shared protocol of detailed neurological examination, neurophysiological examination, cerebrospinal fluid examination, and neuroradiological imaging, as well as clinical follow-up during and after hospitalization. Adult patients (> 18 years) admitted to the participating units during the period between March 1 and April 30, 2020, diagnosed with GBS according to clinical findings and the Brighton Collaboration GBS Working group criteria [7] and COVID-19 were included in this study. Neurophysiology was performed according to Rajabally criteria [10].

A confirmed case of COVID-19 was defined as a positive *SARS-CoV-2* real-time reverse transcriptase PCR (RT-PCR) assay result on a nasopharyngeal swab or a positive serological testing (IgM or IgG) concomitant to the development of typical symptoms of COVID-19.

COVID severity was classified according to the COVID-19 severity score [11], which ranges from 1 to 7: 1, not hospitalized with resumption of normal activities; 2, not hospitalized, but unable to resume normal activities; 3, hospitalized, not requiring supplemental oxygen; 4, hospitalized, requiring supplemental oxygen; 5, hospitalized, requiring nasal high-flow oxygen therapy, noninvasive mechanical ventilation, or both; 6, hospitalized, requiring ECMO, invasive mechanical ventilation, or both; 7, death. Patients were stratified into mild/moderate (1–4 score) and severe (5–6 score).

Clinical course at follow-up was classified according to the modified Rankin scale (mRS) into favorable (0–1), stable (2), and clinical worsening (> 2).

### Standard protocol approvals, registrations, and patient consents

The study received the ethical approval (reference number: Comitato Etico Brianza, session of 10/9/2020). A case report form (CRF) was sent to Italian Neurologists of 20 hospitals.

### Data availability

Anonymized data will be made available on request from any qualified investigator. No deidentified patient data or study-related documents will be shared.

### Statistical analysis

Incidence rate was calculated by dividing the number of cases and time period by the COVID-19 population estimates in Lombardia and by the fraction of population deemed to be positive as of seroprevalence study. These information were obtained by the National Institute of Statistics (ISTAT). To assess the incidence of GBS and 95% confidence intervals in COVID-19 patients we used the following program; https://www2.ccrb.cuhk.edu.hk/stat/confidence%20interval/CI%20for%20single%20rate.htm#Example.

We also obtained information by Regione Lombardia on disease codes of discharge from hospital on the number of cases of GBS diagnosed during the study period (1/3–30/4) in 2019, 2020, and 2021 to compare disease incidence before and after COVID-19 pandemic.

## Results

### Demographic and clinical characteristics of patients with COVID-GBS

**Fig. 1:**
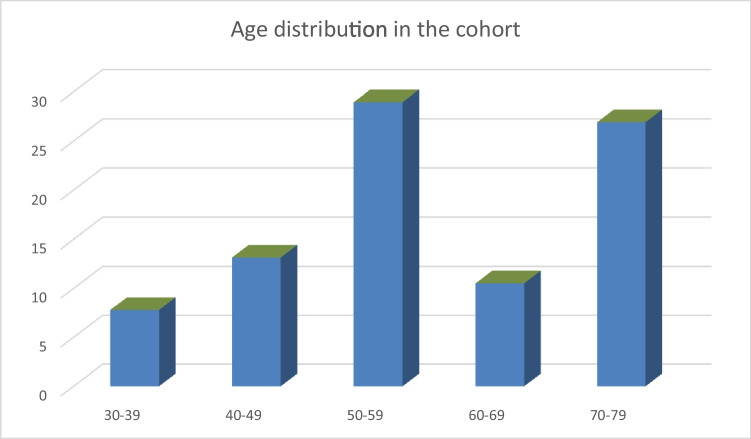
Age distribution in the cohort

Overall, 38 patients were enrolled (Table 1). Mean age was 60.7 years, age distribution by decades is shown in Fig. 1, and 86.8% were male. The cases of COVID-GBS were 18 in the province of Bergamo; 4 in the territory of Crema; 3 in Milan; 2 in Lodi; 2 in Brescia; 2 in Varese; 2 in Pavia; and 1 in Monza, Como, Lecco, Gallarate, and Vizzolo Predabissi. Data concerning 17 patients from Bergamo Hospital, 4 from Crema, 2 from Poliambulanza Hospital in Brescia, 2 from Mondino Institute in Pavia, and 1 from Humanitas Hospital in Milan Province have been reported in previous papers [7, 12].

**Table 1:** Clinical features of COVID-GBS patients

Data (*n* = 38)	Value
Age at onset	60.7 years
Male	89.4%
COVID-19 swab test positivity	33/38
Time interval COVID-19 and GBS	15.1 days
*Symptoms*	
Lower limbs paresis	73.7%
Involvement of upper limbs	55.3%
Tetraparesis	31.6%
Facial nerve involvement	55.3%
Sensory loss and paresthesia	36.8
Hypo-areflexia	100%
Miller-Fisher syndrome	5.26%
*COVID-19*	
Severe	68.4%
Mild/moderate	31.6%
*CSF (n* = *18)*	
Albuminocytological dissociation	71.4%
SARS-COV-2 negative	15/15
*Electrophysiological findings*	33/38
Reduced nerve conduction velocity (NCV)	65.7%
Altered/absent ***F***-wave	82.8%
Increased distal latency	60%
Conduction blocks	5.2%
*Diagnosis*	
Acute inflammatory demyelinating polyneuropathy (AIDP)	81.8%
Acute motor and sensory axonal neuropathy (AMSAN)	12.1%
Acute motor axonal neuropathy (AMAN)	6.0%
*MRI lumbosacral spinal cord with gadolinium*	
Increase in size of lumbosacral roots	2/14
*Treatment*	
Intravenous immunoglobulin (IVIg)	29/38
Plasma exchange (PE)	2/38
IVIg and plasma exchange	2/38
Untreated	5/38
*Clinical course at follow-up*	
Favorable	76.3%
Stable	10.5%
Worsening	13.2%
Death	8.1%
*Respiratory involvement*	
Orotracheal intubation and invasive ventilation	52.6%
*COVID-19 consequences*	
No detectable pulmonary complications	26.3%
Minor pulmonary complications	21%
Severe with a bilateral pulmonary involvement with respiratory distress	52.6%
*Used therapy*	
Hydroxychloroquine	63.1%
Tocilizumab	15.8%
Lopinavir/ritonavir	18.4%
Azathioprine	5.3%
Steroids	44.7%
Prophylactic anticoagulant with LMWH	84.2%

*GBS*, Guillan-Barrè syndrome; *LMWH*, low-molecular weight heparin

All patients performed nasopharyngeal swab test for COVID-19 which was positive in 89.4% of patients, and the 4 patients who tested negative had typical symptomatology and were positive on the serological test.

In 4 patients, it was not possible to ascertain the date of onset of COVID-19 infection, while in 34 the mean interval between COVID-19 onset and GBS onset was 15.1 days. In 5 cases (13.1%), GBS and COVID-19 diagnosis were done simultaneously, while in 6 cases GBS had a late onset ranging from 33 to 45 days after COVID-19 onset.

Concerning clinical symptomatology, 28 patients (73.7%) displayed significant lower limb paresis, in 21 cases (55.3%) an involvement of upper limbs occurred later, evolving into tetraparesis in 12 (31.6%). Facial nerve involvement occurred in 55.3% of cases, and 36.8% suffered from sensory loss and paresthesia, especially in the distal portion of upper and lower limbs, which occurred concomitantly to motor problems. Hypo-areflexia was present in all cases. Only 2 cases displayed a feature of Miller-Fisher syndrome. In 26 cases (68.4%), GBS developed in patients who had severe COVID-19-related symptomatology, whereas 12 patients had a mild/moderate COVID-19 disease according to the COVID-19 severity score [11].

Cerebrospinal fluid (CSF) examination was performed in 28/38 cases (73.7%) and not done in remaining 10 patients. CSF albuminocytological dissociation was detected in 71.4% of tested cases. PCR for *SARS-CoV-2* was negative in all 15 tested patients.

Electrophysiological findings were available in 33/38 patients. At first evaluation, reduced nerve conduction velocity (NCV) was found in 65.7%, an altered/absent *F*-wave in 82.8%, an increased distal latency in 60%, while conduction blocks were present in 5.2%. Based on these data, and on data performed in subsequent neurophysiological examinations during hospitalization, a diagnosis of acute inflammatory demyelinating polyneuropathy (AIDP) was formulated in 81.8% of patients, of acute motor and sensory axonal neuropathy (AMSAN) in 12.1% and of acute motor axonal neuropathy (AMAN) in 6%.

Brain MRI was performed in 16 cases, and it was normal in all cases except in an 80-year-old male patient with a recent diagnosis of cerebellar stroke, while lumbosacral MRI with gadolinium was done in 14 patients showing in 2 an increase in size of lumbosacral roots. An increase in size of the facial cranial nerve was not found even in patients with consistent clinical symptoms.

As of treatment, 29 patients have been treated with intravenous immunoglobulin (IVIg) at the dosage of 0.4 g/kg/day for 5 days, 2 with 5 sessions of plasma exchange (PE), 2 with PE followed by IVIg, and 5 were left untreated due to paucity of symptoms. Three out of the 4 PE-treated patients had a good functional outcome, while one died. Seventeen patients (44.7%) also received oral steroids between 6 and 12 mg per day for 7 days, and among steroid-treated patients 94.1% had a favorable outcome (mRS: 0–2) and one remained stable (mRS: 3), while in those untreated with steroids 19.9% had a worsening of symptoms (mRS: 4–5) (*p* = 0.48).

Based on follow-up evaluation, overall the course was favorable in 29 patients (mRS: 0–2), stable in 4 (10.5%) (mRS: 3) who were sent to a rehabilitation unit, and 5 (13.1%) worsened (mRS: 4–5), of which 3 died (8.1%) (2 had oro-tracheal intubation and invasive ventilation, and one was in continuous positive airway pressure, CPAP), probably as a consequence of the acute respiratory distress syndrome (ARDS) more than of GBS complication. As a matter of fact, among the patients who died, in one case ARDS was associated with septic shock and in two with renal complications. For these 3 patients, an accurate examination of clinical and electrophysiological data did not detect any deficit of thoracic and diaphragm muscles determined by GBS, and autopsy was not performed. Therefore, we can speculate that the 3 deaths are attributable to systemic complications rather than to neurogenic respiratory failure.

Of the 29 patients who had a favorable outcome, 14 had a modified Rankin scale (mRS) of 1 and 5 had a complete recovery at last examination before hospital discharge. Of the 35 surviving patients, 22.8% were sent home, 74.3% transferred to a rehabilitation unit, and 1 to a long-term institution.

As of respiratory involvement, 20 patients (52.6%) underwent oro-tracheal intubation and invasive ventilation, 12 of whom with a previous use of continuous positive airway pressure (CPAP). In 75% of patients with invasive ventilation, neurological deficit improved between admission to discharge, while two patients died. All apart one of the 18 surviving patients were sent to rehabilitation units to continue respiratory rehabilitation. Of the 18 patients who did not receive invasive ventilation, 77.7% improved, 16.6% remained stable, and 1 died. Half of them were sent to rehabilitation units to continue respiratory rehabilitation, and half discharged at home.

As of COVID-19 pulmonary consequences, 10 had no detectable pulmonary complications, 8 had minor pulmonary residuals, and 20 had severe outcome with a bilateral lung involvement with respiratory distress. As of systemic complications, in 4 patients sepsis occurred, causing death in 1 case. Other complications in COVID-GBS were a myocardial infection in 1 subject and severe acute renal failure in 3, 2 of which died.

As of therapy, hydroxychloroquine was used in 63.1%, tocilizumab in 15.8%, lopinavir/ritonavir in 18.4%, azathioprine in 5.3%, steroids in 44.7%, and prophylactic anticoagulant with LMWH in 84.2%.

### Cumulative incidence of GBS in COVID-19

We calculated the occurrence rate of GBS in COVID-19 patients. Considering that the number of identified seropositive cases in Lombardia were 75.732 on April 30, 2020 (https://www.lombardianotizie.online/coronavirus-in-lombardia-i-dati-di-aprile-2020/), the occurrence rate of COVID-GBS was 0.5 per 1000 COVID-19 infections, but the rate of infected individuals in the general population is largely underestimated given that not all the population was screened for the virus and seropositive individuals were more than those detected. If we assume a fraction of 7.5% of population seropositivity in Lombardia according to a seroprevalence study which was however performed in a later period (May 25 to July 15) (https://www.istat.it/it/archivio/246156), occurrence rate decreases to 0.05 per 1000 COVID-19 infections. We can also measure the incidence of GBS in presumed seropositive COVID-19 cases in the period between the beginning of March and end of April 2020 as being of 25.8 cases/100.000/year (95% CI 20.4–32.6) if we consider potential seropositive cases and of 258.6 cases/100.000/year (95% CI 204.8–326.6) if we consider only detected seropositive cases.

**Fig. 2:**
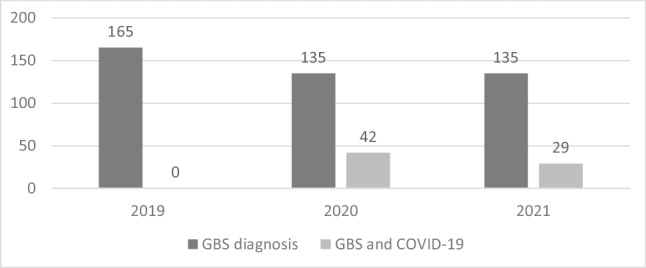
Dismissal codes in 2-month period (March to April) of pre-pandemic period (2019) and pandemic period (2020 and 2021). In black total GBS cases, in gray GBS cases which occurred in COVID-19 positive patients

When we compared the overall incidence of GBS during the same 2-month period of the year (March and April) in Lombardia in the pre-pandemic period (2019) compared to the pandemic period (2020 and 2021), we detected a decrease of the total number of cases of GBS from 165 in 2019 to 135 in 2020 (of whom, 31.1% due to COVID) and 135 in 2021 (of which, 21.5% due to COVID) (Table 2 and Fig. 2). These data probably mean that there was a reduction of GBS cases due to other infections than COVID of 44% in 2020 and of 36% in 2021. As of the number in 2020, it is interesting to notice that our collaborative study was able to retrieve 90% of diagnosed cases (38/42), according to data on code of diagnosis of discharge from hospital obtained from Regione Lombardia (Table 2 and Fig. 2).

**Table 2:** Distribution of dismissal code of diagnosis in 2019, 2020, and 2021

	*2019*		*2020*		*2021*	
*Regional hospital*	*Total hospital discharge*	*Discharge with GBS*	*Total hospital discharge*	*Discharge with COVID-GBS*	*Total hospital discharge*	*Discharge with COVID-GBS*
ASST DEI SPEDALI CIVILI DI BRESCIA	10	7	9	2	8	3
ASST DEI SETTE LAGHI	4	2	1	0	3	1
ASST DEL GARDA	10	9	6	2	5	2
ASST DELLA FRANCIACORTA	5	3	6	3	1	0
ASST DELLA VALCAMONICA					2	0
ASST DELLA VALLE OLONA	4	2	8	1	3	2
ASST DELLA VALTELLINA E DELL'ALTO LARIO	4	2	1	0	4	1
ASST DI BERGAMO EST			5	5	1	0
ASST DI BERGAMO OVEST	14	10	8	3	3	0
ASST DI CREMA	6	3	9	4	2	1
ASST DI CREMONA	2	1			3	0
ASST DI LECCO	3	2	3	1	3	1
ASST DI LODI	1		3	2	2	0
ASST DI MANTOVA	10	7	4	2	7	1
ASST DI MONZA	5	3	3	1	4	1
ASST DI PAVIA	4	3	7	3	15	2
ASST DI VIMERCATE	6	3	1	0	11	3
ASST LARIANA	11	6	11	2	7	2
ASST MELEGNANO E DELLA MARTESANA	9	6	8	1	10	3
ASST NORD MILANO	4	3	4	1	2	0
ASST OVEST MILANESE	10	8	2	0	8	3
ASST PAPA GIOVANNI XXIII	6	3	4	3	7	1
ASST RHODENSE	6	5	4	2	7	1
MILANO	31	23	28	4	17	1
***Total number***	***165***	***111***	***135***	***42***	***135***	***29***

*GBS*, Guillan-Barrè syndrome; *COVID19-GBS*, GBS in COVID-19. In bold the total number

## Discussion

As far as we know, this is the largest case-series of COVID-GBS cases presented so far from a well-defined geographical area, Lombardia, of 10 million people. As a matter of fact, the 38 cases reported have been hospitalized and referred to neurological units, but neurologists from 5 different hospitals reported to have been consulted for GBS patients in other divisions of medicine and pneumology and been unable to follow-up and retrieve clinical charts of such patients unless they were transferred to neurological units. Despite that, according to the data obtained on dismissal diagnostic codes from Regione Lombardia, the 20 neurological units well covered the largest part of Lombardia as they were able to collect 90.4% of cases diagnosed with GBS and COVID. Consequently, the final incidence calculation is higher than those reported by a previous paper with which there is a partial overlap of patients including the same region and a second Northern region, Veneto [12]. The same group published a more recent paper including data from 8 provinces from Northern Italy, confirming a higher incidence of GBS cases during the pandemic period [13]. Even if this study cannot be considered as a population-based study, we can claim that the clinical description of the cohort and the incidence calculation are reliable and partially generalizable to the general population.

All GBS cases were diagnosed according to clinical findings and the Brighton Collaboration GBS Working group criteria [7], and a diagnosis of COVID-19 was defined as a positive *SARS-CoV-2* real-time reverse transcriptase PCR (RT-PCR) assay result on a nasopharyngeal swab or a positive serological testing (IgM or IgG) in a little fraction of patients.

Concerning age distribution of patients in our cohort, results are similar to those reported by Ellul [14] in a review of 19 cases, by Abu-Rumeileh [9] in 73 patients and by Zito [15] on COVID-GBS cases, with some overlap across the three studies, as well as by Filosto [13]. We have to mention that we excluded from our study pediatric cases, since if present they would have been followed in pediatric units or in pediatric neurological units.

It is well-known since first reports on Wuhan cases of COVID-19 that male patients display a more severe disease course and suffer from higher mortality than women [16]. A difference in prevalence between male and female in GBS cases is present in Abu-Rumeileh’s paper [9] (68.5%), Ellul’s paper [14] (68.43%) and Filosto’s paper (74.6%) [13]; similar data have been reported in parainfectious GBS after Zika virus infection [17]. However, our cohort is the one with the highest proportion of male patients, 86.8%. Reasons of these differences can be the existence of hypothetical variants in viral strains of *SARS-CoV-2* occurring in Lombardia, or differences of immunogenetic background across populations. It is worthwhile to mention that such a striking difference in gender ratio was not observed in other neurological complications of COVID-19 cases reported in Lombardia. For example, in a study from the Hospital Papa Giovanni XXIII in Bergamo, 66% of patients with neurological complications were males [18], similar to 61.3% found in another study performed in France [2, 19].

Symptoms occurred with a mean delay of 15.1 days from onset of COVID-19 infection in our patients; however, due to the presence of 6 cases with delayed onset, it must be remarked that a significant number of cases displayed GBS onset in close temporal proximity to COVID-19. Such early onset has been reported previously [20, 21], sometimes it poses diagnostic challenges, and it resembles what happens in other cohorts of COVID-19 cases [9, 12] and in cases of GBS following other infections [4, 5]. Symptoms and signs of GBS in our cohort also did not differ from what is known in GBS following other infections or other series of GBS in COVID-19, apart from the occurrence of facial nerve involvement which was more frequently reported in our patients (55.3% vs. 21% and 16%) [8, 9].

CSF albuminocytological dissociation has been reported in the literature to range from 84.6% [8] to 76% [22], 71.2% [9], and 61.2% [13], a value very close to 71.4% found in our study. Search for *SARS-CoV-2* genome in the CSF yielded negative results in all our 19 patients investigated, as in 31 patients reported by Abu-Rumeileh [9] and in the 5 cases by Toscano [7]. These negative results strongly support the lack of a direct invasion of the virus into the CSF, and that an immune-mediated mechanism is more likely to be responsible of the pathogenesis of COVID-19-related GBS.

Neurophysiological data confirmed a strong prevalence of 81.3% of AIDP, in agreement with Abu-Rumeileh (77.4%) [9], Ellul (66.6%) [14], Zito (68.4%) [15], and Filosto (76.2%) [13].

In our patients, intravenous immunoglobulin and plasma exchange (PE) were found to be effective, further supporting the dysimmune nature of GBS. Concomitant steroid treatment, administered to almost half of COVID-GBS patients, was associated to a more frequent improvement of GBS signs/symptoms compared to those untreated with steroids. There is consolidated evidence that steroids are not effective in GBS either alone [23] or in combination with IVIg or plasma exchange [24–27], but its use in COVID-19 might be considered in the context of several factors, such as the ability of tuning down an ongoing immune reaction and to reduce the pulmonary and systemic complications induced by the virus. Its efficacy in COVID-19 patients was demonstrated by the RECOVERY trial, which demonstrated that oral or intravenous dexamethasone (at a dose of 6 mg once daily for up to 10 days) causes a significant reduction of 28-day mortality among those who were receiving either invasive mechanical ventilation or oxygen alone at randomization [28]. In our cohort, five patients were not treated due to paucity of symptoms according to available guidelines [23, 27].

The course of GBS was slightly better compared to what is known in the literature, with 76.3% who had a favorable outcome in our study compared to 65.3% of patients reported by Hasan [8]. In the same review, however, in-hospital mortality was 3.8% compared to 8.1% found in our cohort of patients. Mortality was 10% in those with invasive ventilation and 5% in those without, much lower than the one reported by Richardson [29] for patients with invasive ventilation during acute pulmonary infection and who did not have GBS. On the contrary, the proportion of patients with neurological improvement was not different, being 75% in those with invasive ventilation and 77% in those who did not, despite the fact that those admitted to invasive ventilation remained immobilized for a long time and were more likely to develop muscle atrophy and critical illness disease. Due to the limited number of reported COVID-GBS cases in the literature, it is difficult to perform a comparison of mortality across GBS patients in COVID-19 compared to other infections, but mortality due to GBS by any other etiology is in a range between 3 and 7%, lower than the one found in this cohort [30].

Our data further supports a causative link between COVID-19 infection and GBS. Incidence of the disease is known to be between 0.9 and 1.9 cases per 100.000 per year [31], figures which are lower than the ones shown in this paper in COVID-19 patients. Our figures are higher than the ones reported in a paper in UK (0.5 cases per 1000 COVID-19 infections in our study vs. 0.016 cases reported in the paper) [32]. Our figures are also greater than the incidence detected in cases of *C. jejuni* (0.25–0.65 per 1000 cases) [33] and Zika virus infection (0.24 per 1000 cases) [4], but lower compared to cases of primary *Cytomegalovirus* (0.6–2.2 per 1000 cases) [34]. Similarly, results point to a stronger link when compared with the other Italian study [12]. Differently from that study, we decided to calculate COVID-GBS incidence by estimating the fraction of population who was seropositive at that time based on available data on seroprevalence.

In a large recent study on 2,005,280 cases of *SARS-CoV-2* positive tests followed for 28 days [35], an excess number of 145 cases of GBS was estimated per 10,000,000 in the 4 weeks following positivity detection; if we extrapolate to our population, even considering that our time of data collection spans 2 months, the number of excess cases expected in the population of Lombardia is likely to be in the order of only a few cases overall.

As of data on dismissal registry reported in Table 2 and Fig. 2, which showed a reduction of GBS cases in 2020 and 2021 compared to 2019, it is important to highlight that the use of containment measures for infections during COVID outbreak, including social distancing, wearing mask, and hand washing among others, not only prevented and controlled infections due to respiratory pathogens other than *SARS-CoV-2* [36], but also other immune-mediated disease like GBS.

There are clear limitations of the study. Data collection was organized by the Neuroscience society (SNO), and, while including 20 units, an underreporting could happen, as opposed to a systematic collection through a national database as happens in the UK [32]. However, data are in line with dismissal registry from hospital, with 90.4% cases detected. Moreover, pediatric patients were not collected in this study.

These results support the need in a patient who presents with COVID-19 to perform a focused neurological examination to identify concomitant GBS. On the other hand, when patients during the COVID-19 pandemic develop signs and symptoms suggestive of GBS, we have to consider them at high risk of being infected with Coronavirus. Moreover, our data also support the beneficial use of steroids in COVID19-GBS, even if more controlled studies are needed.
